# A high-throughput method for genotyping S-RNase alleles in apple

**DOI:** 10.1007/s11032-016-0448-0

**Published:** 2016-02-19

**Authors:** Bjarne Larsen, Marian Ørgaard, Torben Bo Toldam-Andersen, Carsten Pedersen

**Affiliations:** Department of Plant and Environmental Sciences, University of Copenhagen, Thorvaldsensvej 40, 3rd Floor, 1871 Frederiksberg C, Denmark

**Keywords:** S-RNase alleles, Apple, *Malus domestica*, Breeding, Compatibility, Fragment analysis

## Abstract

We present a new efficient screening tool for detection of S-alleles in apple. The protocol using general and multiplexed primers for PCR reaction and fragment detection on an automatized capillary DNA sequencer exposed a higher number of alleles than any previous studies. Analysis of alleles is made on basis of three individual fragment sizes making the allele interpretation highly accurate. The method was employed to genotype 432 *Malus* accessions and exposed 25 different S-alleles in a selection of *Malus domestica* cultivars of mainly Danish origin (402 accessions) as well as a selection of other *Malus* species (30 accessions). The allele S3 (28 %) was the most common among the Danish cultivars followed by S1 and S7 (both 27 %). The alleles S36 and S40 not previously reported from *M*. *domestica* were found in 6 and 17 cultivars, respectively. Complete allelic composition was found in 91 % of the 369 diploid accessions and in 86 % of the 63 triploids concerned. We further identified a relatively high frequency of S33 and S34, which has not been considered by most previous studies. The protocol presented here is easy to adopt and saves both time and work effort compared to previous methods. The robustness is illustrated by the great accuracy and a high number of S-alleles presented.

**Electronic supplementary material:**

The online version of this article (doi:10.1007/s11032-016-0448-0) contains supplementary material, which is available to authorized users.

## Introduction

Self-incompatibility in apple (*Malus* sp.) is controlled by an S-locus with a number of self-incompatibility alleles (Kobel et al. 1939). In this S-RNase-based gametophytic self-incompatibility system, pollen is able to inhibit stigmatic S-RNase, except in case of pollen-bearing S-alleles identical to those in the stigma (De Franceschi et al. 2012). Variation in S-alleles among genotypes grown in orchards is crucial to ensure fertilization and thereby a stable fruit yield. Although small-fruited *Malus* species often are used as pollinators in commercial orchards, compatibility among apple (*M. domestica* Borkh.) cultivars is often important in private gardens. Also in breeding programmes where selection for traits linked to specific S-alleles may occur, there is a risk that certain S-alleles becomes more frequent along generations and that incompatibility problems can be faced.

Incompatibility studies among cultivars have traditionally been carried out by cross-pollination experiments which are highly time consuming and labour expensive. Obtained data from such studies can further be difficult to interpret since it can be difficult to discriminate between full-compatible and semi-compatible genotypes. Molecular studies have been carried out using either allele-specific primers to amplify the S-RNase allele (Broothaerts 2003; Broothaerts et al. 2004; Dreesen et al. 2010; Kim et al. 2006, 2009; Matsumoto and Kitahara 2000; Nybom et al. 2008) or allele-specific restriction enzymes after PCR amplification with universal primers as in the CAPS assay (Kim et al. 2009; Matsumoto and Kitahara 2000). In these studies the DNA fragment sizes were detected on an agarose gel. The CAPS assay requires a rather large selection of restriction enzymes and many equivalent reactions. Allele-specific primers likewise require one reaction per allele, and a large number of PCR reactions should be performed which costs time and money. Furthermore, such studies only expose alleles which were considered at the design and selection phase of appropriate primers; this procedure may prevent possibilities for detection of non-considered alleles present in the gene pool. Finally, conventional fragment base-pair length estimations on agarose gels give an approximate size and do not permit elusive identification of some S-RNase alleles.

Here we present a new high-throughput protocol for detection of S-RNase alleles using general and multiplexed primers, common restriction enzymes and accurate fragment length detection. The fragment analysis pipeline is fully equivalent to that of SSR-markers and thus easy to adopt. As proof of concept, we present the results of genotyping 432 apple genotypes, covering the Danish apple (*Malus domestica*) cultivar collection (334 cultivars) together with a number of mainly European cultivars as well as selections of other *Malus* species, especially *Malus sieversii* and *M. sylvestris*.

## Materials and methods

### Plant material

Plant material was obtained from the gene bank collection at The Pometum (University of Copenhagen, Taastrup, Denmark). Fresh leaf material was collected from vigorously growing shoots and freeze-dried for 48 h. DNA was extracted using DNeasy Plant Mini Kit (Qiagen, Hilden, Germany), following the manufacturers protocol. Samples were diluted to the concentration 2 ng/µL.

### DNA amplification

We combined the general forward primer ASPF3 (Kim et al. 2006) with the new reverse primer EIIWPN-R (Table 1) to amplify most of the described S-RNase sequences. The forward primer always contained the M13-tail for fluorescence detection according to Schuelke (2000). This primer combination was found to be the best for amplifying most S-RNase sequences after testing the forward primers ASPF3, PSNKNGP-F and HGLWPS-F in all combinations with the reverse primers EIIWPN-R, KQNVSEI-R and ASPR3S (Table 1; supplementary file S1). For S-RNase gene fragments yielding a length of more than 1000 bp as well as fragments with unique annealing sites, we designed reverse primers to amplify S3, S5, S10, S39 and S47 (S3/S5/S10-R) and furthermore a specific reverse primer to amplify S16 (S16-R) and S25 (S25-R), respectively. These three reverse primers were used in a multiplex reaction with ASPF3 as forward primer. For S8 a specific forward (S8-F) was developed with two T’s in the 3-prime end specific for the S8-sequence and with the M13-tail for fluorescence detection. The reverse (S8-R) primer was developed for the hypervariable region before the intron position and also specific for the S8-sequence (Table 1; supplementary file S1). All new primers were developed based on sequence alignments as shown in supplementary file S1 using Primer3Plus (Untergasser et al. 2007) and subsequently tested for efficient amplification.

**Table 1:** Primers for S-RNase allele amplification used in this study

Primer	Primer sequence	Amplified S-RNase alleles	Combine primer with	PCR protocol
Reverse primers				
EIIWPN-R	ACGTTYGGCCAAATAATWDCC	S1, S2, S4, S6, S7, S9, S11, S20, S21, S23, S24, S26, S28, S31, S33, S34, S36, S40	ASPF3-F	A
S3/S5/S10-R	TGTTTTGAATYGAAAATTARTTAGGAGT	S3, S5, S10, S39, S47	ASPF3-F	B
S16-R	TGGAAGAGGGCAATTTTGG	S16	ASPF3-F	B
S25-R	TGAAAATGGCTGAAAAACTTTG	S25	ASPF3-F	B
S8-R	ATTTAAGGTTGTTTCTTTGCAATAC	S8	S8-F	B
Forward primers^a^				
ASPF3-F	(M13)-CAATTTACGCAGCARTATCAG			
S8-F	(M13)-TACGATTATTTTCAATTTACGCTT			

^a^The forward primers contained the M13-tail: CACGACGTTGTAAAACGAC

PCR amplification was carried out in a thermal cycler in 20 µL reaction mixture containing 10 ng DNA, 1× Key Buffer (10× Key Buffer, VWR^®^ International, Radnor, Pennsylvania, USA), 1.25 mM MgCl_2_, 0.2 µM dNTP, 0.05 µM forward primer, 0.4 µM reverse primer, 0.25 µM M13 fluorochrome-labelled M13 primer and 0.5 units VWR^®^*Taq* polymerase. The three primers S3/S5/S10-R, S16-R and S25-R were used in a multiplex reaction with ASPF3 as forward primer. The multiplex reaction was carried out in a reaction mixture identical to the one described above, although using 0.2 µM of each of the three reverse primers. During amplification, the products was labelled with FAM, VIC or NED (Schuelke 2000), using the fluorochrome-labelled M13 primer (CACGACGTTGTAAAACGAC) that fits the amplification products and was incorporated in the product during amplification.

PCR program A (Table 1) was initiated with 2 min at 94 °C, and thereafter 18 “touchdown” cycles of 1 min at 94 °C, 30 s at 62 °C for the first cycle and decreasing with 0.5 °C for each cycle and 3 min at 72 °C, afterwards 20 cycles at 94 °C for 1 min, 53 °C for 1 min, 72 °C for 3 min, and then finally 72 °C for 10 min and storage at 4 °C.

PCR program B (Table 1) started with 2 min at 94 °C, followed by 33 cycles of 94 °C for 20 s, 58 °C for 20 s, 72 °C for 2 min and finally 72 °C for 5 min before storage at 4 °C.

### Fragment digestion

All amplification products were analysed both undigested and after being digested with either *Rsa*I or *Taq*^α^I (New England Biolabs, Ipswich, MA, USA). The two restriction enzymes were selected as being suitable for discriminating between the S-alleles (Table 2) after in silico analysis comparing various restriction enzymes. However, products amplified with the primer set S8-F and S8-R were not digested since the primer pair was designed to only amplify the S8-locus, and digestion was therefore not necessary to interpret results. We mixed 2 µL PCR product with 1× CutSmart^®^ Buffer and 10 units restriction enzyme. Digestion with *Rsa*I was carried out for 3 h at 37 °C followed by 65 °C for 20 min before storage at 4 °C. Digestion with *Taq*^α^I was carried out at 65 °C for 3 h followed by 80 °C for 20 min and storage at 4 °C.

**Table 2:** Fragment lengths after amplification with the primers given in Table 1 and digestion with restriction enzymes *RsaI* and *Taq* ^α^I. Calculated and observed fragment lengths are given. Indicated values are inclusive the 19 bp length of the fluorescent M13 tail. Where nd indicated the length that was not determined. ‘Reference cultivar’ indicates the cultivar from which the GenBank sequences are obtained, and ‘Sequenced sample’ is cultivar we sequenced to confirm the allele sequence

Allele	Fragment length						GenBank accession	Reference cultivar	Sequenced sample
	Undigested		*Rsa*I digested		*Taq*I digested				
	Expected	Observed	Expected	Observed	Expected	Observed			
General S-RNase allele primers: ASPF3-F + EIIWPN-R									
S1	560	559–562	223	119–222	125	120–124	EU427454.1	Fuji	Adams Pearmain
S2	369	370–372	369	370–371	130	127–129	DQ219464.1	*M. domestica*	Prima
S3	1513	nd	210	nd	262	nd	EU427455.1	Elstar	
S4	359	361–362	295	294–296	326	328–330	EU427456.1	Gloster	Fejø Æble
S5	1381	1023–1032	423	424–426	371	369–272	EU427460.1	Elstar	
S6	389	390–392	389	390–392	389	390–392	EU427461.1	Citron d’Hiver	*M.sieversii* GS 03-11
S7	340	340–342	340	340–341	125	122–124	EU427457.1	Idared	Pigeon Ildrød
S8	nd	nd	nd	nd	nd	nd	AY744080.1	Ontario mRNA!	
S9	366	367–369	366	367–369	366	367–369	AB270792.1	Florina	Dumelow
S10	1940	nd	412	nd	898	nd	AB428428.1	Maypole	
S11	394	395–398	221	218–220	152	149–152	FJ008669.1	*M. domestica*	Guldborg
S16	2813	nd	967	nd	166	nd	AB428429.1	Maypole	
S20	533	535–537	225	220–222	125	121–123	EU427458.1	Indo	Tønnes, Gravensteiner
S21	396	398–399	64	57–59	363	366–368	AB094494.1	Ribston Pippin	
S23	369	367–368	369	367–368	21	233–234	AF239809.1	Granny Smith	
S24	556	556–558	444	446–448	125	122–124	HQ693065.1	Harangalma	
S25	2582	nd	625	nd	1523	nd	AB428431.1	McIntosh	
S26	379	381	379	381	379	381	EU427459.1	David	Ulderupæble
S28	391	392–394	227	223–225	391	392–394	HQ693074.1	Gyogyi piros	Citronæble, Ørdings Æble
S29	448	nd	193	nd	415	nd	AY039702.1	Anna	
S31	493	496–497	223	220–221	125	120–124	DQ135990.1	York Imperial	Langt Rødt Hindbæræble
S32	373	nd	64	nd	373	nd	DQ135991.1	Burgundy	
S33	960	946-948	569	565-567	960	947–948	AB540121.1	*M. sieversii*	Vejløæble
S34	1040	977–980	622	618–620	1040	976–977	AB540122.1	Adersleber Calville	Spiseæble fra Vejle
S36	399	402–403	64	57–58	366	370–371	EU419865.1	*M. sylvestris*	Ydunsæble
S37	386	nd	227	nd	386	nd	EU419864.1	*M. sylvestris*	
S38	383	nd	227	nd	350	nd	EU419863.1	*M. sylvestris*	
S39	1658	nd	445	nd	725	nd	EU419871.1	*M. sylvestris*	
S40	367	368–370	303	301–304	334	336–338	EU419869.1	*M. sylvestris*	Boiken
S42	2025	nd	604	nd	2025	nd	EU427453.1	Murray	
S44_syl_	934	nd	567	nd	934	nd	EU419862.1	*M. sylvestris*	
S44_dom_	396	nd	64	nd	363	nd	FJ008673.1	*M. domestica*	
S45_syl_	1364	nd	405	nd	1080	nd	EU419861.1	*M. sylvestris*	
S45_sou_	534	nd	224	nd	502	nd	FJ008671.1	*M. soulardii*	
S46_syl_	692	nd	221	nd	659	nd	EU419860.1	*M. sylvestris*	
S46_sou_	365	nd	365	nd	365	nd	FJ008672.1	*M. soulardii*	
S47_syl_	1307	nd	422	nd	172	nd	EU419859.1	*M. sylvestris*	

Allele	Undigested		*Rsa*I digested		*Taq*I digested		GenBank accession	Reference cultivar	Sequenced sample
	Expected	Observed	Expected	Observed	Expected	Observed			
Allele-specific primer assays: ASPF3-F + S3/S5/S10-R, S16-R and S25-R									
S3	423	425–427	210	207–209	264	260–264	EU427455.1	Elstar	Guldspi, Mutsu
S5	399	401–403	399	401–402	273	269–273	EU427460.1	Elstar	Cox’s Orange
S10	380	382–383	380	381–383	380	382–383	AB428428.1	Maypole	Pigeon Ildrød
S16a	787	nd	787	nd	168	nd	AB428429.1	Maypole	
S16b	469	469–470	469	469–470	168	165–166	AB428430.1	Alkmene	Bismarck, Laxton’s Superb
S16c	480	482	480	482	168	166	AB126322.1	Bohnapfel	Ondrup Moseæble
S25	490	493	490	493	490	493–494	AB428431.1	McIntosh	C. J. Hansen 603
S39	417	nd	417	nd	417	nd	EU419871.1	*M. sylvestris*	
S47	388	nd	388	nd	174	nd	EU419859.1	*M. sylvestris*	
Allele-specific primer: S8-F + S8-R									
S8	181	178–179	76	nd	131	nd	AY744080.1	Ontario mRNA!	Bødkeræble, Gadeskovæble, James Grieve, Signe Tillisch

### Fragment length analysis

Three products with different fluorochrome labels were mixed and diluted 12 times before 2 µL was added to a final solution of 12 µL loading buffer (1 mL 0.1× TE-buffer and 40 µL of an internal ROX size standard ranging from 58 to 948 bp). For each cultivar both undigested PCR products and *Taq*^α^I- and *Rsa*I-digested products were analysed. Fragment lengths were detected by ABI 3130xl DNA analyser (Applied Biosystems, Foster City, CA, USA). Analysis and determination of band sizes were performed by the software GeneMarker^®^ v. 2.2.0 (SoftGenetics^®^ LLC, State College, PA, USA), based on an internal ROX-labelled size standard added to each sample. Each band was subsequently checked manually.

The expected restriction fragment lengths of published S-RNase alleles from GenBank accessions (http://www.ncbi.nlm.nih.gov/genbank/) were calculated using CLC main workbench (CLC bio).

### Sequencing

Selected samples were sequenced at GATC Biotech (Germany) after purification with Exonuclease I and Antarctic phosphatase (New England Biolabs). The sequences were compared with accessions of known S-RNase sequences at GenBank to confirm the identifications made based on fragment lengths. Reference cultivars were for example ‘Adam’s Pearmain’ (S1, S3), ‘Golden Delicious’ (S2, S3), ‘Gravensteiner’ (S4, S11, S20), ‘James Grieve’ (S5, S8), ‘Jonathan’ (S7, S9), ‘Cox’s Orange’ (S5, S9), ‘Prima’ (S2, S10), ‘Laxton’s Superb’ (S5, S16b), ‘Ribston (S1, S9, S21), ‘Granny Smith’ (S3, S23), ‘Discovery’ (S1, S24) and ‘Red Delicious’ (S9, S28). To confirm the results, we sequenced one sample for each S-RNase allele, and in all cases we found consensus between the allele we expected and identified.

### Ploidy level

Ploidy levels given in Supplementary file 2 were determined by Larsen et al. (unpublished). For the great majority of accessions, including all Danish cultivars, determination was done by flow cytometry; for remaining accessions, ploidy level was determined from the number of alleles in 15 SSR loci.

## Results

### A new high-throughput protocol for S-RNase allele genotyping

Initially we downloaded and aligned all available different S-RNase allele sequences from *M. domestica* and *M. sylvestris* in GenBank. Based on this we designed new primers and selected suitable restriction enzymes for discriminating all alleles (supplementary file S1). Genotyping of S-RNase alleles was done on basis of fragment lengths of both undigested fragments as well as fragments digested with *Rsa*I and *Taq*^α^I restriction enzymes recognizing GTAC and TCGA, respectively. Only the fragment from the labelled forward primer to the first restriction site is detected on the sequencer simplifying the allele scoring. Calculated and observed fragment lengths are given in Table 2. The nomenclature of S-haplotypes follows the most recent of Matsumoto (2013).

The estimation of fragment sizes using the DNA sequencer and the GeneMarker software differed from the calculated fragment sizes with ±1 or 2 bp as indicated with the differences between ‘expected’ and ‘observed’ fragment lengths (Table 2). However, it was precise enough to draw unambiguous conclusions in each case. There are some S-RNase alleles, which are so similar that data from both *Rsa*I and *Taq*^α^I digestion are needed such as S2 and S23, where both undigested PCR products are 369 bp long, and since there are no *Rsa*I-sites, the *Taq*^α^I digestion is necessary for discrimination.

The S8-allele has been neglected in most studies probably because it is not amplified with the most common general S-RNase primers due to two SNPs in the 3′-end of the ASPF3F annealing sequence. Because of that (Dreesen et al. 2010) developed S8-allele-specific primers, which unfortunately did not work well under our conditions. The forward primer we developed has two unique T’s in the 3′-end, and the reverse primer is placed in the hypervariable region before the intron and is also specific for the S8-sequence (supplementary file S1). This allowed us to identify the S8-allele in 35 Danish cultivars.

### Diversity in S-RNase alleles

**Fig. 1:**
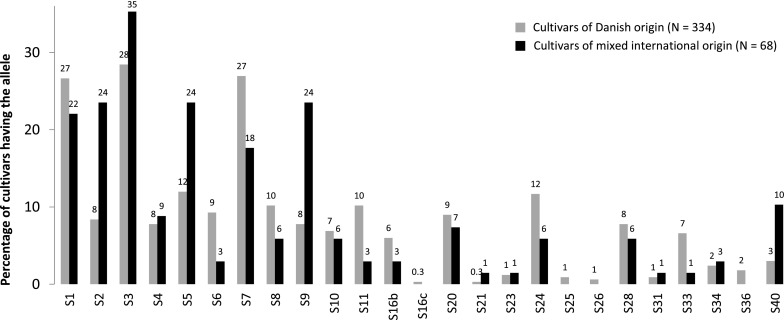
Relative frequency of S-RNase alleles among studied cultivars

Twenty-five different alleles were found among 432 accessions analysed (supplementary file S2). We analysed 369 diploid accessions of which we identified two S-RNase alleles in 91 % of the samples. For the 63 triploid samples, we recognized at least two alleles in all samples and three alleles in 86 % of the accessions. The most common alleles in the Danish cultivars were S1, S3 and S7 which were present in more than 25 % of the cultivars (Fig. 1). Also for the cultivars of mixed international origin considered, S3 (35 %) was the most common allele followed by S1, S5 and S9 (all >22 %).

## Discussion

### High-accuracy prediction of S-RNase alleles

A new protocol for detection and genotyping of S-RNase alleles in apple is presented. It relies on a universal primer pair flanking the variable exon and hypervariable intron areas. Here, variation is detected either directly as length polymorphism of undigested products or after digestion with selected restriction enzymes recognizing polymorphic sites or creating unique restriction fragments. We predicted S-RNase alleles on basis of three different fragment lengths: undigested, *Rsa*I-digested and *Taq*^α^I-digested PCR products. We developed a new general reverse primer placed in a conserved sequence of the second exon to amplify shorter fragments. Some S-RNase alleles with large introns still have large amplicons, which are less efficiently amplified, so we developed three new reverse primers for S3, S5, S10, S16, S25, S39 and S47 for multiplexing PCR. Fragments are sized with high accuracy on a capillary sequencer, and fragments with almost identical lengths such as 391, 394 and 396 bp which were the calculated fragment lengths of S28, S11 and S2, respectively, were more or less distinguishable though the exact identification was done after digestion with two restriction enzymes, where much larger length polymorphisms were obtained. These were after *Rsa*I digestion, 227, 221 and 64 bp, respectively, and 391, 152 and 363 bp, respectively, after *Taq*^α^I digestion.

Even though there was a small discrepancy of 1–3 bp between the calculated fragment lengths and detected fragment lengths, it was possible to make unambiguous interpretations of the obtained results. Such discrepancies of a few base pairs are common, especially between results obtained in different laboratories due to different equipment used. It is probably the relative discrepancies which do not influence interpretation of the results, as long as they are taken into consideration.

The genotyping approach presented here for apple can easily be applied in other species such as pears and cherries. The protocol is very similar to that for SSR-markers, except for the inclusion of a restriction digestion step, making it is easy to adopt and implement in a SSR-marker laboratory.

### Efficiency of genotyping

This protocol provide a reduced work load as three PCR reactions including general and specific primers were able to amplify 25 alleles. In comparison, to identify the same number of alleles by specific primers, 25 PCR reactions would have been needed. Even though our protocol involves an additional step of digesting PCR products with two restriction enzymes before analysis on a capillary sequencer, the method still implies a rationalization compared to previous methods in which analysis is made on individual agarose gels for each allele. Since the use of different fluorescence labelling of products enabled us to multiplex tree products, only one run on the capillary sequencer on average is needed per studied accession.

In studies were specific primers have been used, only a secection of primers have been considered which potentially leaves alleles that are rare or unexpected in the population to remain unexposed. Universal primers as used here, on the other hand, facilitate exposure of unexpected or unknown alleles. None the less, we still lack to expose one allele in a smaller part of the accessions studied here (9 % of the diploids and 14 % of the triploids). A reason for this could be that PCR reaction favours amplification of some alleles, causing less efficiently amplified alleles to be sheltered, when present together. This effect may be extraordinary strong in triploids, where amplification may favour two alleles leaving the third allele not to be exposed. However, this is provided only if genotypes are heterozygous in the S-RNase allele locus, and it might be eventhough it is very rare, that some individuals descends from self-pollination and consequently could be homozygous.

We were able to identify 25 alleles among 432 accessions. In comparison, Nybom et al. (2008) reported 14 alleles, Broothaerts et al. (2004) 18 alleles, Halasz et al. (2011) 20 alleles and only Kim et al. (2009) reporting 22 alleles recorded alleles above S28. In the most recent revision of S-RNase alleles, Matsumoto (2013) lists 27 S-haplotypes, with S34 as the highest number. We identified 22 of these S-haplotypes in the Danish cultivar collection, in addition to S36 and S40. We did not identify S15 and S18 described by Bošković and Tobutt (1999) which nucleotide sequences are not reported (Broothaerts 2003). Further studies should reveal whether these are actually unique alleles or synonyms for other published alleles, by studying ‘Kaiserapfel’ and ‘Menzauer Jägerapfel’ from where the two alleles respectively were described. We did not either find S30 described from the Chinese native *Malus transitoria* (Batalin) C.K. Schneid., or find S29 or S32 which were described from the Israeli cultivar ‘Anna’ and the American cultivar ‘Burgundy’, respectively. However, since the later cultivars have their origin outside Europe and only have been grown very little, if ever, in Denmark; it is not likely that they have contributed genetically to the Danish cultivars.

### Nomenclatural inconsistency

In 2010 Long et al. described and named three S-RNase alleles: S44 from *Malus domestica* and S45 and S46 from *M. soulardii*. Later the same year, the three names were used again by Dreesen et al. (2010) who described several new S-RNase alleles from *M. sylvestris* under the names S36–S47. Whether these S-RNases-sequences have unique functions is not yet known since some of them are rather similar to previously described S-RNases. Here, we followed the nomenclature of Matsumoto (2013) where the alleles were not considered, and until a proper nomenclature has been sorted out, we will use their given names with an abbreviation of the species name as appendix, so that S44_dom_ is different from S44_syl_ (Table 2).

### Rare S-RNase alleles among Danish cultivars

The rarest alleles among the Danish cultivars were S16c and S21, which were only found in ‘Ondrup Moseæble’ and ‘Rød Melba’, respectively. The S16c allele has previously only been reported from Bohnapfel (Bošković and Tobutt 1999) and S21 are also present in ‘Ribston’.

Other rare alleles were S23 and S25 which most probably are inherited from the Australian ‘Granny Smith’ (S3, S23) and the Canadian ‘McIntosh’ (S10, S25) which on a worldwide scale has been widely grown but has played a minor role in Northern Europe. Eight Danish cultivars had S34 which probably descends from the English ‘Cox’s Pomona’ and ‘Queen’ of which the first has been most commonly grown in Denmark and probably the main provider of the allele. S36 and S40 were previously described only from *Malus sylvestris*, but we identified it in a selection of *M. domestica* cultivars as well as a selection of *M. sylvestris* of Danish origin.

### Diversity of S-RNase alleles in Danish apple cultivars

The three most common alleles, S1, S3 and S7, were more than twice as frequent as any other allele. On basis of S-RNase allele frequency among the Danish cultivars, we can divide the alleles into three groups: the common alleles S1, S3 and S7 (>27 % of the cultivars), followed by S2, S4, S5, S6, S8, S9, S10, S11, S16b, S20, S24, S28, S33 (6–12 %) and the rare alleles S16c, S21, S23, S25, S26, S31, S34, S36, S40 (<3 %). In 13 % of the cultivars the genotype was constituted exclusively of the three ‘common’ alleles, and at least one of the three alleles was represented in 68 % of the cultivars. Similar to our findings, S3 and S7 were the two most common alleles found by Nybom et al. (2008) and Halasz et al. (2011) studying cultivars from primarily Northern Europe and the Carpathian basin, respectively.

Among European, American and Japanese cultivars, Broothaerts et al. (2004) also found the most frequent allele to be S3, followed by S2 and S9. Dreesen et al. (2010) again found the most shared allele to be S3 followed by S1 and S5, whereas Matsumoto et al. (2007) found S1, S7 and S9 to be most common. Compared to the two later studies, Nybom et al. (2008) similar to our findings found a low frequency of S9. This was explained by the fact that ‘Cox’s Orange’, ‘Delicious’ and ‘Fuji’ all having S9 are ancestors to many American, South European and Japanese cultivars, but have had a minor impact on cultivars grown in Northern Europe. Concerning ‘Delicious’ and ‘Fuji’, the reason is obviously lack of cultivation in Denmark; however, ‘Cox’s Orange’ has been the most widely planted cultivar in Denmark during the majority of the twentieth century. From SSR-marker analysis (Larsen et al., unpublished), we found ‘Cox’s Orange’ among the most frequent identified parents of the Danish cultivars; however, the great majority of these offspring bears the S5 allele from Cox’s Orange.

According to Pedersen (1950), the main cultivars in Denmark in the 1930s were ‘Belle de Boskoop’ (S2, S3, S5), ‘Bramley’ (S3, S10, S40), ‘Boiken’ (S3, S40), ‘Cox’s Orange’ (S5, S9), ‘Cox’s Pomona’ (S1, S34), Filippa (S7, S24), ‘Gravensteiner’ (S4, S11, S20), ‘Bellefleur de France’ (S2, S7, S20), ‘Ildrød Pigeon’ (S7, S10), ‘Pederstrup’ (S1, S20) and ‘Transparante Blanche’ (S1). Among these, we find a high frequency of the most common alleles identified in the Danish cultivar collection. We found a relatively high presence of S4 (8 %) compared to both Broothaerts et al. (2004) and Halasz et al. (2011) who found S4 to be among the absolute rarest alleles. We also found a relatively high frequency of S11 (10 %) among the Danish cultivars compared to the cultivars of mixed international origin considered. The allele S8 was present in 10 % of the Danish cultivars, probably inherited mainly from ‘James Grieve’.

So far, S33 has only been reported from *Malus sieversii* (Ledeb.) M. Roem. and *M. orientalis* Uglitzk. The allele was described together with S34 by Matsumoto et al. (2010), and the alleles have unfortunately not been considered in most previous studies (Broothaerts et al. 2004; Halasz et al. 2011; Nybom et al. 2008). However, we found S33 in 22 Danish cultivars as well as in the English cultivar ‘Beauty of Kent’. This cultivar has been grown in Denmark since around 1850 and the allele probably is inherited mainly here from.

Cultivars used as parents in breeding programmes differ around the world due to cultural preferences in taste, climate hardiness, etc. According to Laurens (1996), common cultivars in worldwide breeding programmes have to large extent been ‘Braeburn’ (S9, S24), ‘Fuji’ (S1, S9), ‘Gala’ (S2, S5), ‘Golden Delicious’ (S2, S3), ‘Granny Smith’ (S3, S23), ‘Idared’ (S3, S7), ‘Jonathan’ (S7, S9) and ‘Red Delicious’ (S9, S28). Their S-RNase alleles, except S23, are also the most common among the selection of apple cultivars of mixed international origin examined here (Fig. 1), but whether this is a coincidence is not sure. However, it shows that relatively few S-RNase alleles are common among many major cultivars. Among the Danish cultivars, we found a considerable diversity in S-RNase alleles. Breeding programmes should utilize this beautiful diversity and include breeding partners with some of the rarer S-RNase alleles, in oder to prevent potential incompability problems.

## Electronic supplementary material

Below is the link to the electronic supplementary material.

Supplementary file S1. Positions of primer sequences on aligned S-RNase sequences. (PDF 125 kb)

Supplementary file S2. S-RNase alleles identified among 334 Danish apple (*Malus domestica*) cultivars, 68 cultivars of mainly European origin as well as a selection of other *Malus* species. (PDF 98 kb)

Supplementary file S3. Selected reference chromatograms of undigested PCR products. Products amplified with general PCR primers ASPF3-F + EIIWPN-R: ‘Bellefleur de France’ 342 bp (S7), 371 bp (S2), 537 bp (S20); ‘Ribston’ 368 bp (S9), 399 bp (S21), 561 bp (S1); ‘Transperante Rouge’ 391 bp (S6); ‘Gloster’ 369 bp (S40), 393 bp (S28); ‘Holsteiner Cox’ 361 bp (S4), 368 bp (S9); ‘Linnaeus apple from Stenbrohult’ 496 bp (S31); ‘Discovery’ 558 bp (S24), 561 bp (S1). Products amplified with allele-specific, multiplexed primers ASPF3-F + S3/S5/S10-R, S16-R, S25R: ‘Bismarck’ 402 bp (S5), 470 bp (S16b); ‘Bramley’ 383 bp (S10), 426 bp (S3). Product amplified with S8-F + S8-R: ‘James Grieve’ 179 bp (S8). (PDF 105 kb)
