# Supplementary material for: A high-throughput method for genotyping S-RNase alleles in apple
Source: Mol Breed. 2016 Feb 19;36:24. doi: 10.1007/s11032-016-0448-0 (PMC4760992; doi:10.1007/s11032-016-0448-0)
Supplement: Supplementary file 1 — Supplementary file S1. Positions of primer sequences on aligned S-RNase sequences. (PDF 125 kb) [file 11032_2016_448_MOESM1_ESM.pdf]

Supplementary file S1. Positions of primer sequences on aligned S-RNase allele sequences.

Sequence alignment of selections of S-RNase alleles with primer-positions and a consensus peptide sequence below. The first page show part of the region encoding the conserved N-terminal part before the intron-position. The second page show the region encoding the conserved C-terminal part after the intron-position. The sequences for the revers primers are complementary to the indicated concensus-sequences shown. All alignments done with MultAlin: http://multalin.toulouse.inra.fr/multalin/multalin.html

ASPF3 CAATTTACGCAGCARTATCAG

HGLWPS-F AYGGTTTGTGGCCTTCA

PSNKNGP-F CCTTCAAACDNNDWWGGAVVTGA

```
                                             30        40        50        60        70        80                         110       120       130
S1            CAATTTACGCAGCAATATCAGCCGGCTGTCTGCAACTCTAATCCAACTCCTTGTAAGGATCCTCCTGACAAGTTGTTTACCGTTCACGGTTTGTGGCCTTCAAACTCGAATGGAAATGACCCAGAATATT
S24           AATTTTACGCAGCAATATCAGCCGGCTGTCTGCAACTCTAATCCAACTCCTTGTAAGGATCCTACTGACAAGTTGTTTACTGTTCACGGTTTGTGGCCTTCAAACTCGAATGGAAATGACCCAAAATATT
S20           CAATTTACGCAGCAATATCAGCCGGCTGTCTGCCACTCTAATCCAACTCCTTGTAAGGATCCTCCTGACAAGTTGTTTACTGTTCACGGGTTGTGGCCTTCAGACTCGAATGGAAATGACCCAAAATATT
S31           CAATTTACGCAGCAATATCAGCCGGCTGCCTGCAACTCTAATCCAACTCCTTGTAAGGATCCTCCTGACAAGTTGTTCACTGTTCACGGTTTGTGGCCTTCAGACTCGAATGGAAATGACCCAAAATATT
S29           CAATTTACGCAGCAATATCAGCCGGCTGTCTGCAACTTTACTGCTACTCCTTGTAAGGATCCTCCTGACAAGTTGTTTACGGTTCACGGTTTGTGGCCTTCAAACGCGAAGGGAAATGACCCAGAAGGTT
S3            CAATTTACGCAGCAATATCAGCCGGCTGTCTGCAGCTCTAATCCTACTCCTTGTAAGGATCCTCCTGACAAGTTGTTTACGGTTCATGGTTTGTGGCCTTCAAATGTTAATGGAAGTGACCCCAAGAAAT
S39           CAATTTACGCAGCAATATCAGCCGGCTGCCTGCAACTCTAACCCTACTCCTTGTAACGATCCTACTGACAAGTTGTTTACGGTTCACGGATTGTGGCCTTCAAAAGTTAAAGGTCCTGACCCACACGACT
S10           CAATTTACGCAGCAGTATCAGCCGGCTGTCTGCAACTCTAATCCTACTCCTTGTAAGGATCCTCCGGACAAGTTGTTTACGGTTCATGGTTTGTGGCCTTCAAATGTTAATGGAAGTGACCCCAAGAAAT
S16a/S16b/S22 CAATTTACGCAGCAATATCAGCCGGCTGTCTGCAACTTTAATCCTACTCCATGTAAGGATCCTACTGACAAGTTGTTTACGGTTCACGGTTTGTGGCCTTCAAACAACGTAGGAGGTGACCCAGAATCTT
S16c          CAATTTACGCAGCAATATCAGCCGGCTGTCTGCAACTTTAATCCTACTCCATGTAAGGATCCTACTGACAAGTTGTTTACGGTTCACGGTTTGTGGCCTTCAAACAACGTAGGAGGTGACCCAGAATCTT
S25           CAATTTACGCATCAATATCAGCCGGCTGTCTGCAACTCTAATCGTACTCCTTGTAAGGATCCTCCTGACAAATTGTTTACGGTTCACGGTTTGTGGCCTTCAAACAGGAATGGACCTGACCCAGAGTATT
S5            CAATTTACGCAGCAATATCAGCCGGCTGCCTGCAAGTTTCACCATACTCCTTGTAAGGATCCTCCTGACAAGTTGTTTACGGTTCACGGATTGTGGCCTTCAAACTTTAATGGACCTGACCCAGAAAACT
S45           CAATTTACGCAGCAATATCAGCCGGCCGTATGCAACTCTAATCCTACTCCTTGTAACGATCCTCCTGACAAGTTGTTTACAGTTCATGGTTTGTGGCCTTCAAACAAGAATGGACCTGACCCAGAAAAAT
S47           CAATTTACGCAGCAATATCAGCCGGCTGTCTGCAACTCTAATCCTCCTCCTTGTAAGGATCCTCCTGACAAGTTGTTTACGGTTCATGGTCTGTGGCCTTCAAATGTTAATGGAAGTGACCCCAAGAAAT
S33           CAATTTACGCAGCAATATCAGCCGGCTGCCTGCAACTCTAATCCTACTCCTTGTAAGGATCCTCCTGAGAAATTGTTTACGGTTCACGGTTTGTGGCCTTCAAACTCTAATGGACCTGACCCAGTAAACT
S34           CAATTTACGCAGCAATATCAGCCGGCAGTCTGCAACTCTAATCCTACTCCTTGTAAGGATCCTACTGAGAAATTGTTTACGGTTCACGGTTTGTGGCCTTCAAACTCTAATGGACCTGACCCGGTAAACT
S44syl        CAATTTACGCAGCAATATCAGCTGGCTGCCTGCAACTCTAATCCTACTCCTTGTAAGGATCCTCCTGAGAAATTGTTTACGGTTCACGGTTTGTGGCCTTCAAACTCTAATGGACCTGACCCAGTAAACT
S46/S54       CAATTTACGCAGCAATATCAGCCGGCTGTCTGCAACTTTAGTTCTACTCCTTGTAAGGATCCTCCTGACAAGTTGTTTACGGTTCACGGTTTGTGGCCTTCAAACGCGAAGGGAAATGACCCAGAAGGTT
S8            CAATTTACGCTTCAATATCAACCGGCTGTCTGCAACTCTAGTCGTACTCCTTGTAAGGATCCTCCTGACAAGTTGTTTACGGTTCACGGTTTGTGGCCTTCGAACAGGAATGCACCTGACCCAGAGTATT
S2            CAATTTACGCAGCAATATCAGCCGGNTGCCTGCNACTCTAATCCTACTCCTTGTAAAGATCCTCCTGACAAGTTGTTTACGGTTCACGGTTTGTGGCCTTCAAACATGAATCGAAGTGAATTATTTAATT
S23           CAATTTACGCAGCAATATCAGCCGGCTGCCTGCAACTCTAATCCTACTCCTTGCAAGGATCCTCCTGCCAAGTTGTTTACGGTTCACGGTTTATGGCCTTCAAACTGGAACTTACCCGACCCAATCTTCT
S26           CAATTTACGCAGCAATATCAGCCGGCTGTCTGCAACTCTAATCCTACCCCTTGTAAAGATCCTCCTGACAAGTTGTTTACGGTTCACGGTTTGTGGCCTTCAAACTCAACAGGACGTGACCCAAAATATT
S6            CAATTTACGCAGCAATATCAGCCGGCCGTATGCAACTCTAATCCTACTCCTTGTAACGATCCTGCTGACAAGTTGTTTACGGTTCACGGTTTGTGGCCTTCAAACAAGAATGGACCTGACCCAGAAAAAT
S28           CATTTTACGCAGCAATATCAGCCGGCCGTATGCAACTCTAATCCTACTCCTTGTAACGATCCTCCTGAAAAATTGTTTACGGTTCACGGTTTGTGGCCTTCAAACAAGAATGGACCTGACCCAGAAAAAT
S37           CAATTTACGCAGCAATATCAGCCAGCTGTCTGCAACTCTAAACCTACTCCTTGTAAGGATCCTCCTGACAAGTTGTTTACGGTTCACGGTTTGTGGCCTTCAAACTTGAATGGACCTCACCCAGAAAATT
S38           CAATTTACGCAGCAATATCAGCCAGCTGTCTGCAGATCTAATCCTACTCCTTGTAAGGATCCTACTGACAAGTTGTTTACGGTTCACGGTTTGTGGCCTTCAAACTTGAATGGACCTCACCCAGCAAATT
S32           CAATTTACGCTGCAATATCAGCCGGCTGTCTGCAACTCTAATGGTACTCCTTGTAAGGATCCTCCTGACAAGCTGTTTACGGTTCACGGTTTGTGGCCTTCAAACTTTTTAGGACCTGACCCAGAGTATT
S4            CAATTTACGCAGCAATATCAGCCGGCTGCCTGTAACTCTAATCCCACTCCTTGTAAGGATCCTACTGACAAGTTGTTTACGGTTCATGGTTTGTGGCCTTCAAACAAAATAGGACGTGACCCAGAATATT
S40           CAATTTACGCAGCAATATCAGCCGGCTGCCTGCAACTCTAATCCTACTCCTTGTAAGGATCCTACTGACAAGTTGTTTACGGTTCACGGTTTGTGGCCTTCAAACAAAATAGGAGGTGACCCGAAATATT
S11           CAATTTACGCAGCAATATCAGCTGGCTGTCTGCAACTCTAATCGTGCTCCTTGTAAGGATCCTCCTGACAAGTTGTTTACGGTTCACGGTTTGTGGCCTTCAAGCATGGTAGGACCTGACCCAAGTAATT
S44dom        CAATTTACGCAGCAATATCAGCTGGCTGTCTGCAACTCTAGTCGTACTCCTTGTAAGGATCCTCCTGACAAGTTGTTTACAGTTCACGGTTTGTGGCCTTCAAGCATGGTAGGACCTGACCCAAGTAATT
S21           CAATTTACGCAGCAATATCAGCTGGCTGTCTGCAACTCTACTCGTACTCCTTGTAAGGATCCTCCGGACAAGTTGTTTACGGTTCACGGTTTGTGGCCTTCAAGCATGGTAGGACCTGACCCAAGTAATT
S36           CAATTTACGCAGCAATATCAGCTGGCTGTCTGCAACTCTAATCGTACTCTTTGTAAGGATCCTCCTGACAAGTTGTTTACGGTTCACGGTTTGTGGCCTTCAAGCATGGTAGGACCTGACCCAAGTAAAT
S7            CAATTTACGCAGCAATATCAGTTGGCTGCCTGCAACTCTAAACCTATTCCTTGTAAGGATCCTCCTGACAAGTTGTTTACGGTTCACGGTTTGTGGCCTTCAGACTCGAATGGACATGACCCAGTAAATT
S9            CAATTTACGCAGCAATATCAGCCGGCTGTCTGCCACTTTAATCCTACTCCTTGTAGAGATCCTCCTGACAAGTTGTTTACGGTTCACGGTTTGTGGCCTTCAAACAGCTCAGGAAATGACCCAATATACT
```

F T Q Q Y Q P A V C N S N P T P C K D P P D K L F T V H G L W P S N K N G P D P

Fig S1

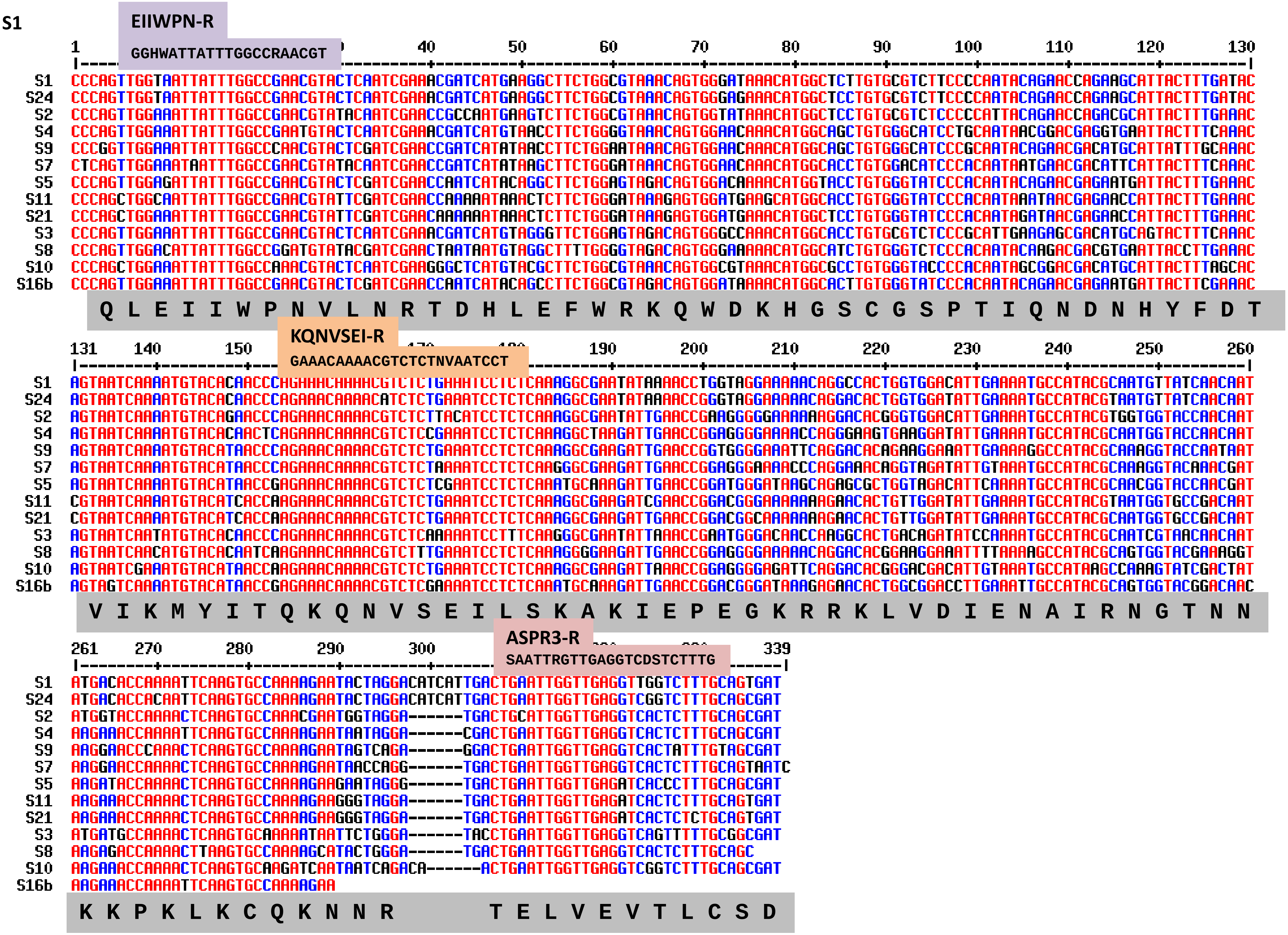
EIIWPN-R
GGHWATTATTTGGCCRAACGT
KQNVSEI-R
GAAACAAAACGTCTCTNVAATCCT
ASPR3-R
SAATTRGTTGAGGTCDSTCTTTG
S1
S24
S2
S4
S9
S7
S5
S11
S21
S3
S8
S10
S16b
Q L E I I W P N V L N R T D H L E F W R K Q W D K H G S C G S P T I Q N D N H Y F D T
V I K M Y I T Q K Q N V S E I L S K A K I E P E G K R R K L V D I E N A I R N G T N N
K K P K L K C Q K N N R T E L V E V T L C S D

Fig S1

Alignment of the S-alleles S3, S5, S10, S25, S39 and S47 with the position of a reverse primers specific for the S-alleles S3, S5, S10, S39, and S47, but not S25 and the S25-R primer specific for S25 only. The exon-intron splice site is marked with a red arrow.

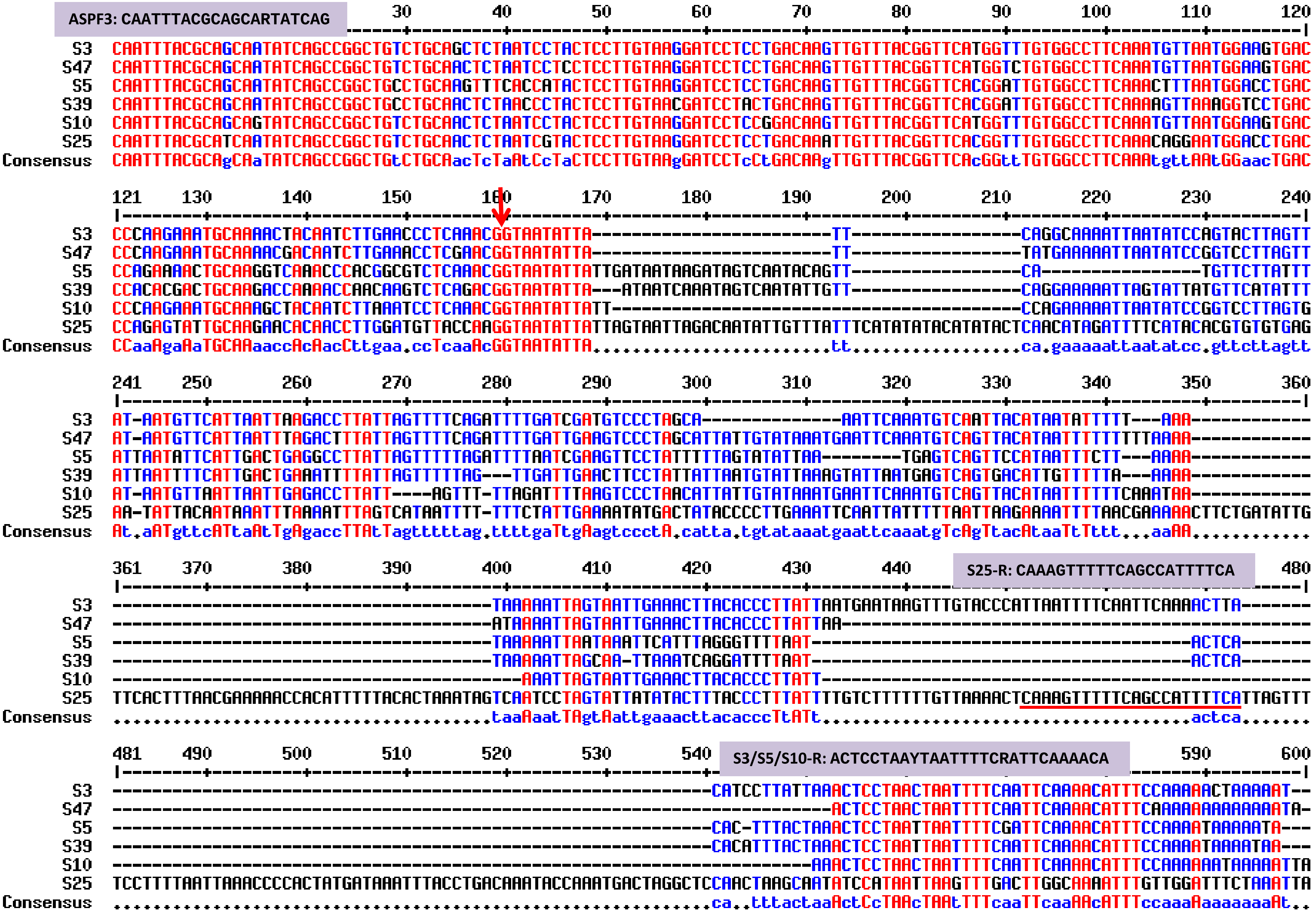

Fig S1

Alignment of the S-alleles S16a, S16b and S16c and the position of a reverse primer specific for these three S16-alleles giving rise to length polymorphisms between them. The exon-intron splice site is marked with a red arrow.

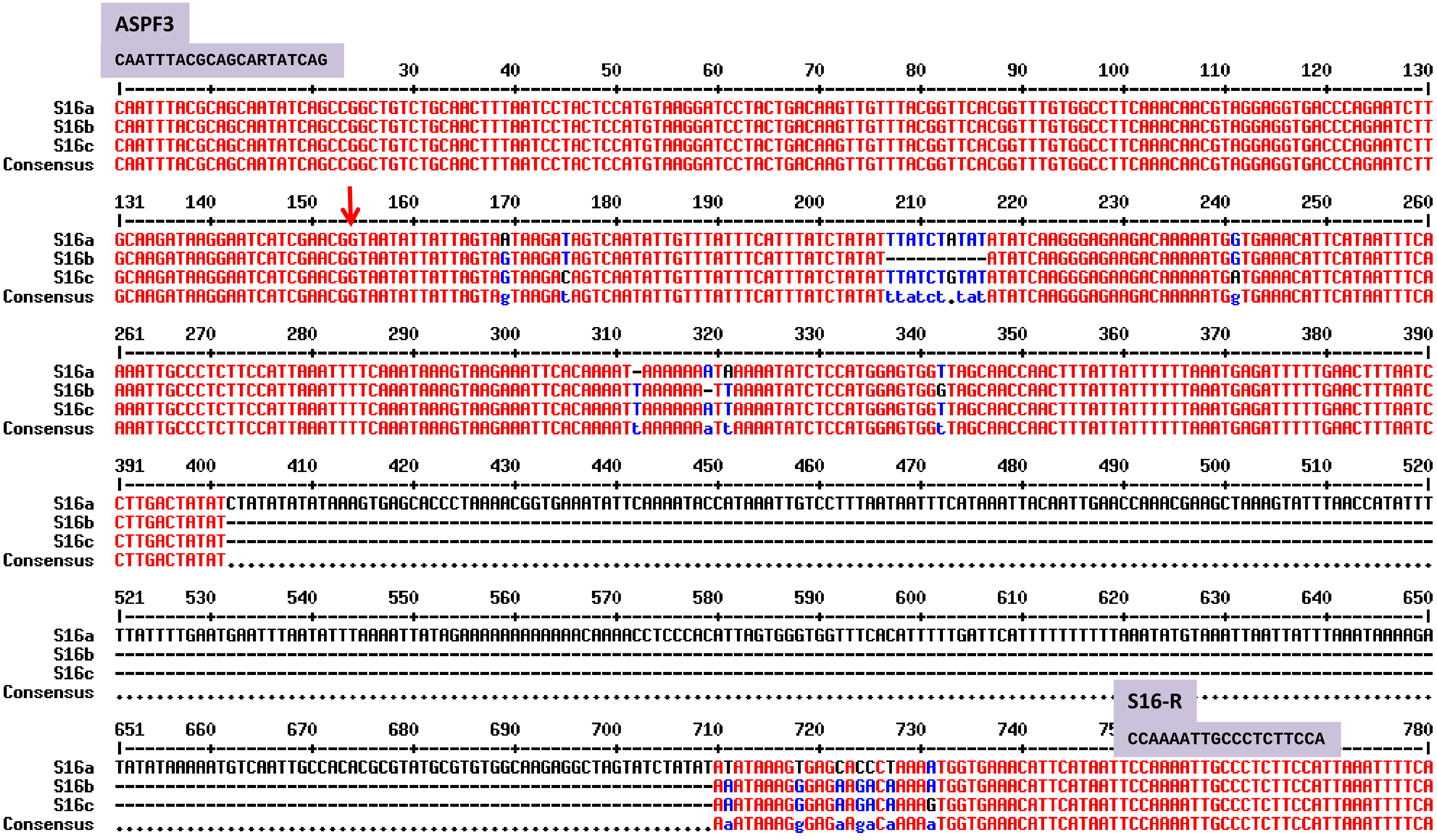

Fig S1

Alignment of S8 with a selection of other S-alleles. The general primer ASPF3 has a mismatch in 3'end of the annealing site on S8 and is therefor not working well, so instead a primer pair with allele-specific 3'-ends for both forward and revers primers was developed.

ASPF3: CAATTTACGCAGCARTATCAG

S8-F: TACGATTATTTTCAATTTACGCTT

```
                                         30        40        50        60        70        80   85
S1                     CAATTTACGCAGCAATATCAGCCGGCTGTCTGCAACTCTAATCCAACTCCTTGTAAGGATCCTCCTGACAAGT
S4                     CAATTTACGCAGCAATATCAGCCGGCTGCCTGTAACTCTAATCCCACTCCTTGTAAGGATCCTACTGACAAGT
S11                    CAATTTACGCAGCAATATCAGCTGGCTGTCTGCAACTCTAATCGTGCTCCTTGTAAGGATCCTCCTGACAAGT
S2                     CAATTTACGCAGCAATATCAGCCGGNTGCCTGCNACTCTAATCCTACTCCTTGTAAAGATCCTCCTGACAAGT
S3                     CAATTTACGCAGCAATATCAGCCGGCTGTCTGCAGCTCTAATCCTACTCCTTGTAAGGATCCTCCTGACAAGT
S6                     CAATTTACGCAGCAATATCAGCCGGCCGTATGCAACTCTAATCCTACTCCTTGTAACGATCCTGCTGACAAGT
S7                     CAATTTACGCAGCAATATCAGTTGGCTGCCTGCAACTCTAAACCTATTCCTTGTAAGGATCCTCCTGACAAGT
S9                     CAATTTACGCAGCAATATCAGCCGGCTGTCTGCCACTTTAATCCTACTCCTTGTAGAGATCCTCCTGACAAGT
S8         TACGATTATTTTCAATTTACGCTTCAATATCAACCGGCTGTCTGCAACTCTAGTCGTACTCCTTGTAAGGATCCTCCTGACAAGT
Consensus              CAATTTACGCAGCAATATCAGccGGctGtcTGcaaCTCTAAtCctacTCCTTGTAAgGATCCTcCTGACAAGT

           86       95        105       115       125                                 …5   170
S1         TGTTTACCGTTCACGGTTTGTGGCCTTCAAACTCGAATGGAAATGACCCAGAATATTGTAAGGCACC------GCCATATCATAC
S4         TGTTTACGGTTCATGGTTTGTGGCCTTCAAACAAAATAGGACGTGACCCAGAATATTGCAAGACAAG------GAATCGTCGGAA
S11        TGTTTACGGTTCACGGTTTGTGGCCTTCAAGCATGGTAGGACCTGACCCAAGTAATTGCTCGATAAG------GAATATTCGGAA
S2         TGTTTACGGTTCACGGTTTGTGGCCTTCAAACATGAATCGAAGTGAATTATTTAATTGCAGTAGTTCAAACGTGACTTATGCGAA
S3         TGTTTACGGTTCATGGTTTGTGGCCTTCAAATGTTAATGGAAGTGACCCCAAGAAATGCAAAACTACAATCTTGAACCCTCAAAC
S6         TGTTTACGGTTCACGGTTTGTGGCCTTCAAACAAGAATGGACCTGACCCAGAAAAATGCAAGACTACAGCCCTGAATTCTCAGAA
S7         TGTTTACGGTTCACGGTTTGTGGCCTTCAGACTCGAATGGACATGACCCAGTAAATTGCAGTAAATCAACCGTGGATGCTCAGAA
S9         TGTTTACGGTTCACGGTTTGTGGCCTTCAAACAGCTCAGGAAATGACCCAATATACTGCAAGAATACAACCATGAATTCTACTAA
S8         TGTTTACGGTTCACGGTTTGTGGCCTTCGAACAGGAATGCACCTGACCCAGAGTATTGCAAAGAAACAACCTTAAATGTTACCAA
Consensus  TGTTTACgGTTCAcGGTTTGTGGCCTTCAaaca.gaatgGAc.TGAcccagaaaAtTGcaaga.aacaa.c.tGaat..Tc.gAa
```

S8-R: GTATTGCAAAGAAACAACCTTAAAT
