# Supplementary material for: A high-throughput method for genotyping S-RNase alleles in apple
Source: Mol Breed. 2016 Feb 19;36:24. doi: 10.1007/s11032-016-0448-0 (PMC4760992; doi:10.1007/s11032-016-0448-0)
Supplement: Supplementary file 2 — Supplementary file S2. S-RNase alleles identified among 334 Danish apple (Malus domestica) cultivars, 68 cultivars of mainly European origin as well as a selection of other Malus species. (PDF 98 kb) [file 11032_2016_448_MOESM2_ESM.pdf]

Acc. no.	Cultivar	Ploidy	S-RNase alleles		
**Danish apple (*M. domestica*) cultivars**					
**247**	Alsisk Citronæble	2N	S3	S33	
**300**	Aminæble	2N	S2	S3	
**260**	Andersæble	2N	S10	S28	
**2**	Anna's Æble	2N	S4	S9	
**301**	Anne Høj Æble	2N	S4	S24	
**3**	Antonius	2N	S1	S16b	
**222**	Apple 207 Knuthenborg	**3N**	S1	S20	S28
**4**	Arreskov	2N	S9	S24	
**274**	Askeæble	2N	S1	S24	
**5**	Augustæble	2N	S9	S24	
**6**	Aunhusæble	2N	S1	S16b	
**236**	Auroavej	2N	S5	S6	
**265**	Bananæble	**3N**	S2	S7	S11
**302**	Bankegårds Grøn Sommeræble	**3N**	S1	S4	S6
**7**	Barritskov Madæble	2N	S4	S20	
**8**	Basnæsæble	**3N**	S1	S7	S20
**9**	Bedstefars Æble	2N	S7	S40	
**303**	Bedstemoræble	2N	S11	S24	
**20**	Bjerlevæble	2N	S8	S33	
**10**	Blangstedgård nr. 156	2N	S5	S7	
**11**	Blankholm	2N	S2	S7	
**12**	Bodil Neergaard	2N	S1	S7	
**13**	Bodils Æble	2N	S3	S11	
**304**	Bogense kålæble	2N	S9	S11	
**305**	Border F	2N	S3	S20	
**14**	Borgherre	**3N**	S1	S3	S4
**278**	Bornholms Reinet	**3N**	S1	S3	S6
**15**	Bredstedæble	2N	S20	S24	
**16**	Bredæble	2N	S4	S8	
**246**	Broholm	2N	S3	S28	
**17**	Broholm Rosenæble	2N	S3	S28	
**18**	Broström Guldreinette	**3N**	S1	S2	S7
**19**	Brændekilde	2N	S1	S8	
**225**	Brøndæble	2N	S8	S11	
**244**	Butteræble	2N	S24	S33	
**306**	Bødker Pigeon	2N	S7	S28	
**21**	Bødkeræble	2N	S8	S11	
**22**	Bøgelundsæble	2N	S6	S8	
**23**	Bøgh's Citronæble	2N	S3	S24	
**203**	C. J. H. 12-32	2N	S1	S2	
**204**	C. J. H. 12-37	2N	S2	S3	
**205**	C. J. H. 14-20	2N	S7	S8	
**206**	C. J. H. 23-13	2N	S5	S10	
**200**	C. J. H. 6-18	2N	S8	S11	
**201**	C. J. H. 7-27	**3N**	S1	S16b	
**202**	C. J. H. 9-31	2N	S5	S10	
**224**	C. J. Hansen 1017	2N	S7	S25	

**223**	C. J. Hansen 603	2N	S7	S25	
**307**	Ceske Æble	2N	S8	S20	
**308**	Charlamowsky	2N	S3	S28	
**24**	Cherry Cox	2N	S5	S9	
**309**	Citronæble	2N	S23	S28	
**25**	Comtesse Johannes Æble	2N	S1	S3	
**27**	Dansk Rosenhäger	2N	S1	S10	
**28**	Dauglykkeæble	2N	S1	S4	
**29**	Degneæble	2N	S1	S8	
**310**	Det Gamle Madæble	2N	S5	S6	
**30**	Diana	2N	S16b	S24	
**31**	Ditte Wiuff	2N	S3	S9	
**311**	Dorthe Margrethe	2N	S3	S5	
**32**	Drejæble	2N	S8	S20	
**33**	Dronning Louise	2N	S7	S9	
**312**	Dumelow	2N	S1	S9	
**241**	Dyndegaardsæble	**3N**	S1	S5	S24
**271**	Dyndegaardsæble Bornholm	**3N**	S1	S5	S24
**213**	Edle Wendelborg	2N	S1	S16b	
**34**	Ejby Æble	2N	S6	S8	
**314**	Elin Andersen	2N	S1	S10	
**315**	Elise Lund	2N	S7	S16b	
**35**	Elmelund	2N	S1	S20	
**36**	Else Lindstow	2N	S1	S5	
**317**	Engelsk Pigeon	2N	S7	S10	
**37**	Ernst Trier	**3N**	S1	S5	S7
**316**	Eva Roth	2N	S1	S26	
**318**	Evan Pippin	2N	S1		
**38**	Farum Æble	**3N**	S3	S5	S28
**39**	Fejø Æble	2N	S4	S5	
**40**	Femø Æble	**3N**	S1	S3	S6
**214**	Ferskenrødt Sommeræble	2N	S3	S8	
**41**	Filippa	2N	S7	S24	
**208**	Filippa Anka	2N	S1	S7	
**125**	Filippa Harritslev	2N	S7	S24	
**42**	Flaskehalser	**3N**	S1	S20	
**43**	Flintinge	**3N**	S1	S5	S24
**44**	Fru Glahns Æble	2N	S2	S7	
**320**	Frueæble	2N	S16b	S24	
**321**	Frøbjergæble	2N	S6	S8	
**45**	Frørups Æble	2N	S7	S24	
**298**	Frøstrups Sommeræble	2N	S7	S9	
**46**	Fuhræble	2N	S7	S24	
**48**	Fynsk udvalg II	2N	S2	S7	
**49**	Fynsk udvalg III	2N	S1	S4	
**51**	Fynsk udvalg V	**3N**	S2	S7	S11
**52**	Fynsk udvalg VI	2N	S4	S8	
**238**	Fåborgæble	2N	S3	S8	
**233**	Fåremule	2N	S3	S10	

**26**	Gadeskovæble	**3N**	S8	S11	S24
**322**	Gammel Strand Æble	2N	S3	S5	
**323**	Generalæble	**3N**	S2	S3	S5
**53**	Glasæble	2N	S1	S4	
**54**	Glostrup Guldreinette	2N	S3	S7	
**269**	Graneli	2N	S20		
**209**	Grannegårdsæble	2N	S3	S33	
**325**	Granvænget	2N	S3	S5	
**55**	Gravenfin	2N	S3	S8	
**60**	Gravensteiner (Gul Graasten)	**3N**	S4	S11	S20
**326**	Grevinde Ahlefeldt	2N	S5	S16b	
**56**	Grænseæble	2N	S7	S8	
**324**	Grøn Vinter Rambour	**3N**	S2	S3	S7
**327**	Grønkær	2N	S1	S11	
**328**	Grønt Sødæble	2N	S3	S5	
**57**	Gråmølleæble	**3N**	S1	S3	S10
**237**	Graasten 69-438	**3N**	S2	S5	S8
**282**	Graasten Fra 1905	2N	S1	S9	
**329**	Gul Kalvil	2N	S3	S6	
**330**	Gul Pigeon	2N	S7	S9	
**331**	Gul Vinter Æble	**3N**	S3	S7	
**67**	Guldborg	2N	S3	S11	
**68**	Guldborg Rød	2N	S3	S11	
**69**	Guldspir	2N	S3	S20	
**70**	Guldæble	2N	S6	S10	
**72**	Hans Mathiesen	2N	S1	S7	
**73**	Hans Wassard	**3N**	S3	S7	
**299**	Harløseæble	2N	S5	S24	
**74**	Harreslev Æble	2N	S3	S7	
**75**	Helsingør Guldreinette	2N	S3	S28	
**76**	Henrik Jensens Æble	2N	S3	S4	
**77**	Herfølge voksæble	2N	S7	S9	
**78**	Herman	2N	S7	S9	
**245**	Herring Pippin	2N	S1	S5	
**79**	Herschendsgave	2N	S9	S40	
**332**	Hes Æble	2N	S7	S33	
**80**	Hillerslev Æble	2N	S1	S34	
**81**	Hillested Æble	2N	S1	S34	
**239**	Hindbæræble	2N	S8	S20	
**83**	Hjortholm	**3N**	S1	S4	S28
**84**	Hofman Bang	2N	S7	S24	
**256**	Holfisæble	2N	S1	S8	
**333**	Hollandsk Reinette	2N	S1	S6	
**85**	Holmeæble	2N	S28	S31	
**86**	Holstenhus	**3N**	S1	S3	S6
**87**	Hornbækæble	**3N**	S2	S3	S33
**335**	Horneskov	2N	S1	S3	
**336**	Hornslevæble	2N	S3	S7	
**212**	Hugonsæble	2N	S3	S6	

**242**	Hullegaardsæble	2N	S28		
**337**	Hvid Astrakan	2N	S3	S11	
**88**	Hvidkilde Voksæble	2N	S3	S9	
**338**	Hvidt Vinteræble	**3N**	S1	S3	S6
**216**	Hyltofteæble	2N	S2	S7	
**90**	Højbjerg Kalvil	2N	S20	S24	
**91**	Højbjerg Sommerkalvil	2N	S20	S28	
**235**	Høje-Taastrup Æble	2N	S6	S7	
**263**	Hønseæble	2N	S3	S33	
**92**	Hørningsholm	2N	S7		
**339**	Høstcitron	2N	S3	S11	
**93**	Høve Reinet	2N	S1	S16b	
**94**	Ildrød Eilstrup	2N	S1	S2	
**340**	Indslev	2N	S5	S36	
**96**	Ingers Æble	2N	S9	S23	
**97**	Ingrid Marie	2N	S5	S34	
**232**	Jacober	2N	S3	S10	
**99**	Jarnak	**3N**	S1	S3	S4
**259**	Julieæble	2N	S2	S5	
**101**	Jølbyæble	2N	S4		
**341**	Kanelæble	2N	S24		
**111**	Kansleræble	2N	S7	S24	
**82**	Karen Blixen	2N	S1	S5	
**342**	Karin Badell	2N	S1	S8	
**343**	Kasvenske Negenose	2N	S7	S16b	
**102**	Kathrineæble	2N	S11	S20	
**228**	Kattehovedæble	2N	S1	S40	
**261**	Kildebækæble	2N	S7		
**103**	Knud Lunn	2N	S3	S40	
**104**	Koldemosegaard	2N	S2	S3	
**105**	Kronprins Frederiks Taffelæble	2N	S7	S24	
**220**	Kundbyæble	2N	S7	S24	
**227**	Kyholm Reinet	2N	S1	S5	
**344**	Kærby Ydun	2N	S23	S36	
**345**	Kålæble	2N	S9	S11	
**47**	Ladbyæble	2N	S1	S7	
**346**	Landsgrav Sommeræble	2N	S3	S8	
**106**	Langeland	**3N**	S2	S7	S11
**50**	Langelandsæble	**3N**	S2	S7	S11
**108**	Langt Rødt Hindbæræble	2N	S10	S31	
**347**	Langå	**3N**	S1	S3	S9
**348**	Langå Æble	**3N**	S1	S3	S9
**277**	Lejle Pluk	2N	S5	S6	
**109**	Lise Legind Æble	2N	S3	S4	
**110**	Ludvigsens Æble	2N	S10	S20	
**112**	Lundbytorpæble	2N	S7	S16b	
**113**	Lunds Doucin	2N	S8	S28	
**114**	Lyngbyæble	2N	S2	S10	
**115**	Løgæble	2N	S3	S40	

**71**	Låsbyæble	2N	S3	S24	
**258**	Maegårdsæble	**3N**	S2	S7	S8
**116**	Maglemer	2N	S6	S11	
**349**	Magtimer	2N	S6	S11	
**118**	Maren Nis	2N	S3	S5	
**350**	Marienborg	2N	S2	S11	
**119**	Marieæble Fra Hjortholm	**3N**	S1	S4	S28
**120**	Markussens Æble	2N	S7	S31	
**121**	Marselisborg Sommeræble	2N	S10	S24	
**122**	Martha	2N	S1	S28	
**123**	Mathilde Æble	2N	S1	S16b	
**351**	Melonæble	2N	S3	S6	
**250**	Miang Æble	**3N**	S1	S3	
**229**	Mikkel Peders Æble	2N	S16b	S23	
**352**	Minne Marie	2N	S5	S40	
**124**	Mormors Æble	2N	S1	S33	
**240**	Mosedeæble	2N	S1	S28	
**251**	Møllers Venus	2N	S3	S33	
**249**	Mølleskov	2N	S7	S24	
**353**	Mølleæble	2N	S1	S20	
**126**	Niels Juul	**3N**	S1	S5	S7
**354**	Niels Morten Æble	2N	S3	S7	
**127**	Niels Olsens Æble	2N	S1	S7	
**128**	Ninas Æble	2N	S8	S16b	
**355**	Nonne tit Bastard	2N	S7	S9	
**129**	Nonnetit Bastard	2N	S7	S9	
**130**	Nonnetit Fra Als	2N	S3	S7	
**294**	Nonnetit Fra Odstrup	2N	S8	S24	
**356**	Nor Æble	2N	S5	S40	
**217**	Nybøllegaard	2N	S7	S10	
**131**	Nørregaardsæble	2N	S1	S7	
**218**	Ondrup Moseæble	2N	S16c		
**132**	Ondrup Sommeræble	2N	S3	S16b	
**133**	Ormslevæble	2N	S2	S28	
**134**	Oudrupgaards Høstæble	2N	S11		
**135**	Pederstrup	2N	S1	S20	
**289**	Pederstrup nr. 417	2N	S3	S11	
**276**	Per Jensker	2N	S3	S33	
**151**	Per Smeds Æble	2N	S4	S33	
**136**	Peter Hansens Æble	2N	S3	S7	
**137**	Pigeon Almindelig	2N	S7	S10	
**142**	Pigeon Fra Juellinge	2N	S7	S11	
**143**	Pigeon Fra Maribo	2N	S7	S11	
**89**	Pigeon Hvid Sommer	2N	S7	S24	
**95**	Pigeon Ildrød	2N	S7	S10	
**141**	Pigeon Ildrød Dronningmølle	2N	S7	S24	
**107**	Pigeon Langeland Hvid	2N	S1	S7	
**144**	Pigeon Rød Vinter	2N	S3	S7	
**145**	Pigeon Spejlsby	2N	S7	S10	

**146**	Pigeon Stribet	2N	S7	S10	
**147**	Pilehaveæble	2N	S1	S24	
**268**	Pingeløkke	2N	S3	S33	
**358**	Pommeræble	2N	S7	S11	
**148**	Prinsesse Anne Marie	2N	S3	S8	
**149**	Prinsesse Benedicte	2N	S3	S8	
**150**	Prinsesse Margrethe	2N	S2	S34	
**296**	Pundsæble	2N	S9	S34	
**152**	Purpurella	2N	S1	S10	
**153**	Pæregaards Æble	2N	S5	S20	
**154**	Rasmus Hansens Æble	2N	S1	S5	
**155**	Rifbjerg Gylling	2N	S4	S9	
**156**	Rifbjerg Skarlagen Pearmain	2N	S3	S7	
**157**	Ringkloster Kammerjunker	2N	S7	S24	
**158**	Risskov Rambour	2N	S7	S11	
**159**	Rolund	2N	S5	S6	
**160**	Rosenholm	2N	S3	S24	
**231**	Rosenæble	2N	S7		
**359**	Rynkeby strib	2N	S11	S40	
**360**	Rød melba	2N	S10	S21	
**287**	Rød Nonnetit nr. 361	2N	S7	S11	
**162**	Rødby Æble	2N	S2	S7	
**163**	Rødhætte Æble	2N	S6	S8	
**215**	Rødt Æble	2N	S7	S33	
**164**	Signe Tillisch	2N	S8	S34	
**165**	Sildig Sandholt Æble	**3N**	S1	S9	S20
**166**	Skarridsø Æble	2N	S1		
**167**	Skenkelsø Æble	**3N**	S3	S6	S33
**168**	Skensved Æble	2N	S5	S7	
**361**	Skomageræble	2N	S3	S24	
**169**	Skovfoged	2N	S3	S7	
**363**	Skør Vinsur	2N	S24	S36	
**267**	Slamra	2N	S3	S33	
**170**	Slesvigsk Jordbæræble	2N	S3	S10	
**364**	Små Grønne	**3N**	S1	S20	
**171**	Soesmark Æble	2N	S3	S4	
**172**	Sofie Æble	**3N**	S1	S3	S7
**366**	Sommercitron	**3N**	S9	S28	S33
**365**	Sommeræble	2N	S3	S5	
**173**	Sorøæble	2N	S7	S16b	
**174**	Spilmoseæble	2N	S1	S2	
**175**	Spiseæble Fra Vejle	2N	S7	S34	
**367**	Spækæble	2N	S28		
**368**	Stabolt	2N	S3	S6	
**266**	Stenberg	2N	S33		
**226**	Stjerneæble nr. 1	2N	S1	S4	
**230**	Stjerneæble nr. 2	2N	S1	S4	
**369**	Stor gul Bourg	2N	S16b	S33	
**176**	Strandby Æble	2N	S20	S24	

**177**	Sukkertop Fra Vaalse	2N	S6	S7	
**178**	Sukkeræble	2N	S1	S36	
**370**	Svanninge	2N	S7	S9	
**179**	Svindingeæble	2N	S7	S20	
**600**	Sybergæble	2N	S3	S25	
**180**	Søbjergæble	**3N**	S16b	S28	S33
**181**	Søde Æbler Fra Aalsrode	2N	S1		
**182**	Sønderskov	2N	S33		
**183**	Tagesminde Æble	2N	S40		
**184**	Terndrup Æble	2N	S7	S20	
**185**	Thoras Æble	2N	S16b		
**186**	Thyrislund	2N	S1	S3	
**371**	Tidlig Richard 11/44	**3N**	S1	S3	S6
**187**	Tjele Æble	2N	S1	S11	
**372**	Tjenerkroner	2N	S6	S36	
**279**	Torslunde	2N	S4	S34	
**210**	Tullebølleæble	2N	S2	S5	
**188**	Tyra Mathiesen	2N	S1	S11	
**189**	Tyregod Kalvil	2N	S1	S28	
**252**	Tyrrestrup	2N	S8	S20	
**190**	Tønnes	2N	S3	S20	
**373**	Tåsinge Gråsten	2N	S3	S5	
**191**	Udby Reinet	2N	S1	S11	
**192**	Udlejre Reinet	2N	S1	S11	
**262**	Uggerløseæble	2N	S6		
**219**	Ulderupæble	2N	S6	S26	
**194**	Vallekilde Sommeræble	2N	S3	S28	
**193**	Vallekilde Æble	**3N**	S1	S7	
**243**	Vejløæble	2N	S3	S33	
**264**	Vindrueæble	2N	S6	S20	
**257**	Vintergråsten	**3N**	S1	S3	S8
**270**	Vinterkalvil	2N	S2	S7	
**195**	Vinteræble Fra Halskov	2N	S3	S40	
**196**	Vinteræble Fra Marebæk	2N	S3	S28	
**221**	Virumgård	**3N**	S3	S4	S28
**197**	Ydunsæble	2N	S5	S36	
**272**	Yndbyæble	2N	S10	S24	
**254**	Æbeltoftæble	2N	S3	S33	
**198**	Ærøæble	2N	S16b	S24	
**199**	Ørdings Æble	**3N**	S3	S4	S28
**375**	Østergård	2N	S6	S20	
**1**	Aabyæble	**3N**	S6	S20	
**Apple (*M. domestica*) cultivars of mixed international origin**					
**401**	Adams Pearmain	2N	S1	S3	
**402**	Alfriston	2N	S7	S11	
**403**	Allington	2N	S1	S5	
**405**	Aroma	2N	S5	S7	
**406**	Beauty of Bath	2N	S1	S4	
**407**	Beauty of Kent	**3N**	S2	S3	S33

**408**	Belle de Boskoop	**3N**	S2	S3	S5
**439**	Bellefleur de France	**3N**	S2	S7	S20
**411**	Bismarck	2N	S5	S16b	
**476**	Bledisloe Cox	2N	S5	S9	
**412**	Blenheim	**3N**	S1	S3	S10
**413**	Boiken	2N	S3	S40	
**414**	Bramley	**3N**	S3	S10	S40
**415**	Casseler Reinet	2N	S2	S3	
**416**	Coulon	**3N**	S1	S2	
**419**	Cox´s Pomona	2N	S1	S34	
**418**	Cox´s Type Nr. I	2N	S5	S9	
**488**	Cox's Orange	2N	S5	S9	
**420**	Danziger Kantæble	2N	S2	S7	
**481**	Discovery	2N	S1	S24	
**425**	Dumelow	2N	S1	S9	
**426**	Early Golden Russet	2N	S4		
**428**	Early Victoria	2N	S4	S8	
**429**	Ecklinville	2N	S4	S10	
**469**	Elstar	2N	S3	S5	
**430**	Frogmore	2N	S3	S24	
**432**	Gladstone	2N	S11	S20	
**468**	Gloster	2N	S28	S40	
**472**	Golden Delicious	2N	S2	S3	
**434**	Grahams Royal Jubilee	2N	S5	S24	
**435**	Granny Smith	2N	S3	S23	
**436**	Grå Fransk Reinet	**3N**	S3	S7	S9
**438**	Gul Richard	2N	S7	S24	
**437**	Guldpearmain	2N	S1	S4	
**478**	Holsteiner Cox	**3N**	S4	S5	S9
**334**	Honeygold	2N	S2	S8	
**440**	James Grieve	2N	S5	S8	
**484**	Jonagold	**3N**	S2	S3	S9
**474**	Jonagored	**3N**	S2	S3	S9
**444**	Jonathan	2N	S7	S9	
**447**	Keswick Codlin	2N	S1	S20	
**448**	Laxton's Superb	2N	S5	S16b	
**479**	Linnaeus apple from Stenbrohult	2N	S3	S31	
**450**	Lord Derby	2N	S1	S9	
**451**	Lord Lambourne	2N	S2	S5	
**452**	Lord Suffield	2N	S20		
**453**	Mutsu	**3N**	S2	S3	S20
**357**	Oriole	2N	S2	S3	
**454**	Orleans Reinet	**3N**	S1	S3	S40
**477**	Pigeon Hvid Vinter	2N	S3	S7	
**473**	Pigeon Oberdicks	2N	S28	S40	
**485**	Prima	2N	S2	S10	
**455**	Prinz Albert von Preussen	2N	S7	S40	
**456**	Queen	2N	S3	S34	
**482**	Queen Cox	2N	S5	S9	

**480**	Rajka	2N	S5	S7	
**422**	Red Delicious	2N	S9	S28	
**457**	Ribston	**3N**	S1	S9	S21
**486**	Ritt Bjerregaard	2N	S5	S9	
**400**	Rød Ananas	2N	S6	S7	
**470**	Schieblers Pigeon	2N	S3	S40	
**460**	Summerred	2N	S2	S9	
**461**	Sweet Delicious	2N	S3	S28	
**462**	Sävstaholm	2N	S1	S7	
**464**	Transparante Blanche	2N	S1		
**465**	Transparante de Croncels	2N	S2	S3	
**466**	Transparante Rouge	2N	S6	S8	
**467**	Wealthy Red	2N	S3	S9	
***Malus* sp.**					
**502**	*Malus × moerlandsii* 'Profusion'	2N	S40		
**501**	*Malus coronaria*	2N	S6		
**500**	*Malus sargentii*	2N	S20		
**505**	*Malus sieboldii* 'Diana'	2N	S6		
**504**	*Malus sieboldii* 'Scarlet'	2N	S6		
**508**	*Malus sieversii* "GB 03-04"	2N	S6	S10	
**526**	*Malus sieversii* "GB 03-05"	2N	S6	S40	
**510**	*Malus sieversii* "GB 03-09"	2N	S1	S9	
**512**	*Malus sieversii* "GB 03-12"	2N	S1	S3	
**513**	*Malus sieversii* "GB 03-13"	2N	S1	S7	
**514**	*Malus sieversii* "GB 03-15"	2N	S16b	S40	
**516**	*Malus sieversii* "GB 06-02"	2N	S24		
**519**	*Malus sieversii* "GB 07-07"	2N	S4	S11	
**520**	*Malus sieversii* "GB 07-09"	2N	S7		
**521**	*Malus sieversii* "GB 08-04"	2N	S4	S7	
**522**	*Malus sieversii* "GB 08-17"	2N	S1	S16b	
**511**	*Malus sieversii* "GS 03-11"	2N	S6		
**515**	*Malus sieversii* "GS 05-06"	2N	S6	S7	
**518**	*Malus sieversii* "GS 07-02"	2N	S7		
**523**	*Malus sieversii* "GS 10-04 F"	2N	S1	S7	
**362**	*Malus* 'Skovtrold'	**3N**	S6	S7	S23
**533**	*Malus sylvestris* "malus 11"	2N	S28	S36	
**541**	*Malus sylvestris* "malus 112"	2N	S40		
**534**	*Malus sylvestris* "malus 25"	2N	S36		
**535**	*Malus sylvestris* "malus 37"	2N	S1	S6	
**536**	*Malus sylvestris* "malus 43"	2N	S8	S20	
**537**	*Malus sylvestris* "malus 57"	2N	S7	S31	
**538**	*Malus sylvestris* "malus 64"	2N	S4	S8	
**539**	*Malus sylvestris* "malus 75"	2N	S6		
**540**	*Malus sylvestris* "malus 85"	2N	S7		
