# Supplementary figures and images for: A high-throughput method for genotyping S-RNase alleles in apple
Source: Mol Breed. 2016 Feb 19;36:24. doi: 10.1007/s11032-016-0448-0 (PMC4760992; doi:10.1007/s11032-016-0448-0)

## Supplementary file S3. Reference chromatograms.

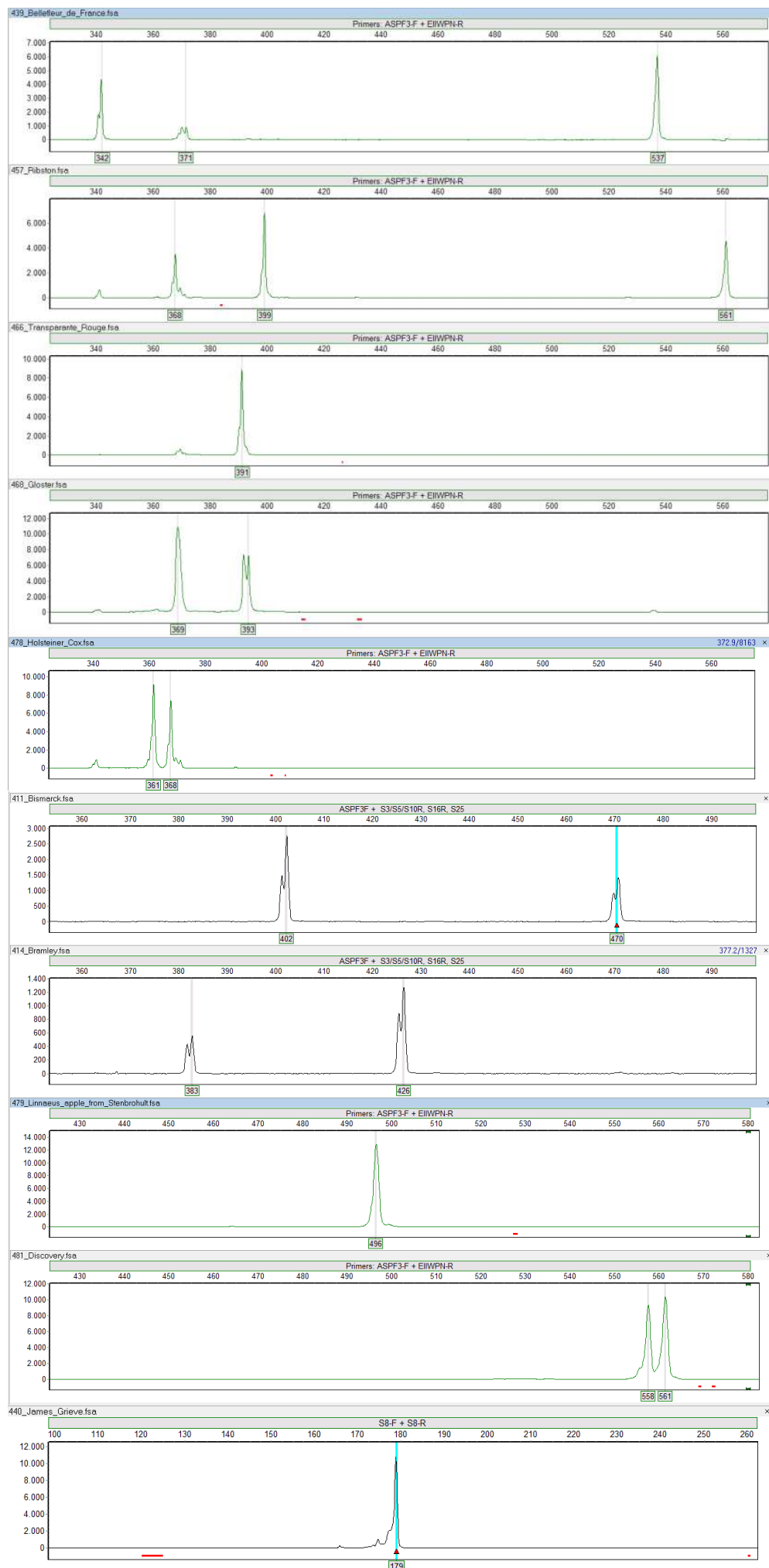

Supplement: Supplementary file 3 — Supplementary file S3. Selected reference chromatograms of undigested PCR products. Products amplified with general PCR primers ASPF3-F + EIIWPN-R: ‘Bellefleur de France’ 342 bp (S7), 371 bp (S2), 537 bp (S20); ‘Ribston’ 368 bp (S9), 399 bp (S21), 561 bp (S1); ‘Transperante Rouge’ 391 bp (S6); ‘Gloster’ 369 bp (S40), 393 bp (S28); ‘Holsteiner Cox’ 361 bp (S4), 368 bp (S9); ‘Linnaeus apple from Stenbrohult’ 496 bp (S31); ‘Discovery’ 558 bp (S24), 561 bp (S1). Products amplified with allele-specific, multiplexed primers ASPF3-F + S3/S5/S10-R, S16-R, S25R: ‘Bismarck’ 402 bp (S5), 470 bp (S16b); ‘Bramley’ 383 bp (S10), 426 bp (S3). Product amplified with S8-F + S8-R: ‘James Grieve’ 179 bp (S8). (PDF 105 kb) [file 11032_2016_448_MOESM3_ESM.pdf]
